# Prediction of the most deleterious non-synonymous SNPs in the human *IL1B* gene: evidence from bioinformatics analyses

**DOI:** 10.1186/s12863-024-01233-x

**Published:** 2024-06-10

**Authors:** Ola Abuzaid, Abeer Babiker Idris, Semih Yılmaz, Einass Babikir Idris, Leena Babiker Idris, Mohamed A. Hassan

**Affiliations:** 1 Faculty of Medicine, Erciyes University, Kayseri, Turkey; 2 Department of Medical Microbiology, Faculty of Medical Laboratory Sciences, University of Khartoum, Khartoum, Sudan; 3 Department of Agricultural Biotechnology, Faculty of Agriculture, Erciyes University, Kayseri, Turkey; 4 Erciyes Teknopark, Promoseed Biotechnology A.Ş, Kayseri, Turkey; 5 Department of Medical Microbiology, Rashid Medical Complex, Riyadh, Saudi Arabia; 6 Psychiatry Resident, Sudan Medical Specialization Board, Khartoum, Sudan; 7 Department of Bioinformatics, Africa City of Technology, Khartoum, Sudan; 8 Sanimed International Lab and Management L.L.C, Abu Dhabi, UAE

**Keywords:** IL1B, nsSNPs, Deleterious SNPs, Cancer-causing nsSNPs, In silico tools

## Abstract

**Background:**

Polymorphisms in *IL1B* play a significant role in depression, multiple inflammatory-associated disorders, and susceptibility to infection. Functional non-synonymous SNPs (nsSNPs) result in changes in the encoded amino acids, potentially leading to structural and functional alterations in the mutant proteins. So far, most genetic studies have concentrated on SNPs located in the *IL1B* promoter region, without addressing nsSNPs and their association with multifactorial diseases. Therefore, this study aimed to explore the impact of deleterious nsSNPs retrieved from the dbSNP database on the structure and functions of the IL1B protein.

**Results:**

Six web servers (SIFT, PolyPhen-2, PROVEAN, SNPs&GO, PHD-SNP, PANTHER) were used to analyze the impact of 222 missense SNPs on the function and structure of IL1B protein. Five novel nsSNPs (E100K, T240I, S53Y, D128Y, and F228S) were found to be deleterious and had a mutational impact on the structure and function of the IL1B protein. The I-mutant v2.0 and MUPro servers predicted that these mutations decreased the stability of the IL1B protein. Additionally, these five mutations were found to be conserved, underscoring their significance in protein structure and function. Three of them (T240I, D128Y, and F228S) were predicted to be cancer-causing nsSNPs. To analyze the behavior of the mutant structures under physiological conditions, we conducted a 50 ns molecular dynamics simulation using the WebGro online tool. Our findings indicate that the mutant values differ from those of the IL1B wild type in terms of RMSD, RMSF, *Rg*, SASA, and the number of hydrogen bonds.

**Conclusions:**

This study provides valuable insights into nsSNPs located in the coding regions of *IL1B*, which lead to direct deleterious effects on the functional and structural aspects of the IL1B protein. Thus, these nsSNPs could be considered significant candidates in the pathogenesis of disorders caused by IL1B dysfunction, contributing to effective drug discovery and the development of precision medications. Thorough research and wet lab experiments are required to verify our findings. Moreover, bioinformatic tools were found valuable in the prediction of deleterious nsSNPs.

**Supplementary Information:**

The online version contains supplementary material available at 10.1186/s12863-024-01233-x.

## Background

Single nucleotide polymorphisms (SNPs) constitute the predominant form of genetic variations in the human genome, accounting for approximately 90%. These variations occur every 100–300 bases and involve single-base pair changes in alleles, with around 500,000 situated within the coding region [1, 2]. The coding-region SNPs, particularly non-synonymous SNPs (nsSNPs), can lead to changes in the amino acids, potentially causing structural and functional modifications in the mutant proteins. The potential adverse effects of nsSNPs encompass a wide range of consequences, including destabilization of protein structures and influencing gene regulation, as well as protein properties and interactions [2, 3]. However, it is important to note that not all nsSNP-induced changes are necessarily harmful [4]. With millions of SNPs in the entire human genome, a primary challenge in planning population-based genotyping studies is detecting SNPs that are likely to impact phenotypic functions and contribute to disease development [5]. Predicting the functional consequences of a nsSNP is based on various attributes of the polymorphism, some of which are determined only by sequence information, such as the types of residues present at the SNP location. Structural attributes such as solvent accessibility can be selected if the protein sequence harboring the nsSNP either has a known 3D structure or closely resembles a protein sequence with a known structure [6]. Therefore, it is important to utilize suitable computational approaches and empirical rules, using probabilistic and machine learning methods to distinguish deleterious and damaging nsSNPs from benign ones.

The significant role of genetics influencing individual susceptibility to inflammatory diseases has been proposed for over four decades. Several genes associated with an increased susceptibility to these complex diseases, including proinflammatory cytokines, have been identified [5, 7, 8]. In this study, we aimed to predict the structural and functional effects of nsSNPs mapped in genetic variants of the human *interleukin-1 beta* (*IL-1B*) gene. IL-1β is a proinflammatory cytokine belonging to the IL-1 family, which collectively spans approximately 430 kb and is clustered on chromosome 2q13–21. The most studied IL-1 genes include *IL-1 A, IL-1B*, and *IL-1RN*, which encode the pro-inflammatory cytokines IL-1α and IL-1β, along with the endogenous IL-1 receptor antagonist (IL-1ra) [9]. IL-l α and β are synthesized as 31-kDa precursors without signal peptides. Processing of IL-lα or IL-1β to mature forms of 17-kDa requires the removal of N-terminal amino acids by specific cellular proteases (i.e., calpain, caspase-1) [10]. On the other hand, IL-1ra has evolved with a signal peptide, allowing it to be easily transported out of cells. IL-1B precursor (proIL-1B) is not fully active and a significant amount is secreted after cleavage by the intracellular protease. Also, its secretion is tightly regulated because of its high potency [11].

IL-1β exerts a wide range of biological effects in various tissues and is involved in inflammatory, metabolic, physiologic, hematopoietic, and immune processes [9]. The presence of functional polymorphisms in cytokine genes can lead to an imbalance in the production of pro- and anti-inflammatory cytokines [12]. Dysregulation in the expression of inflammatory cytokines has been linked to inflammatory lesions, autoimmune diseases, and malignancies [12, 13]. Genetic diseases resulting from polymorphic *IL1B* genetic variants can be attributed to two primary scenarios. The first one is when SNPs in the upstream region of the *IL1B* modify regulatory motifs, such as transcription factor binding sites (TFBSs) which influence the transcriptional regulation and expression of the IL1B protein. Changes in the expression level of the *IL1B* gene affect the extent of the pro-inflammatory response and are associated with various disease phenotypes [9, 14]. The second scenario involves genetic polymorphisms in the coding region of *IL1B*, which may lead to structural or functional alterations in the IL-1B protein when located in essential sites, i.e., receptor binding or biological activities. These polymorphisms can also affect the ability of IL1B to bind to the IL-1 receptor (IL-1R). Consequently, such alterations influence IL-1B-mediated cell signaling pathways and the activation of inflammatory cells [15].

So far, many genetic studies have concentrated on SNPs within the *IL1B* gene promoter region, with no studies focusing on nsSNPs within the coding region. Taking this into account, as well as the fact that IL-1B plays a key role in a variety of infectious and inflammatory diseases, this study aimed to identify the most deleterious nsSNPs in the coding region and to anticipate their structural and functional implications in the IL-1B protein from bioinformatics evidence. This study, for the first time, provided valuable insights into the effects of amino acid variations on IL1B protein structure, function, and disease association.

## Results

### nsSNP retrieval

**Fig. 1:**
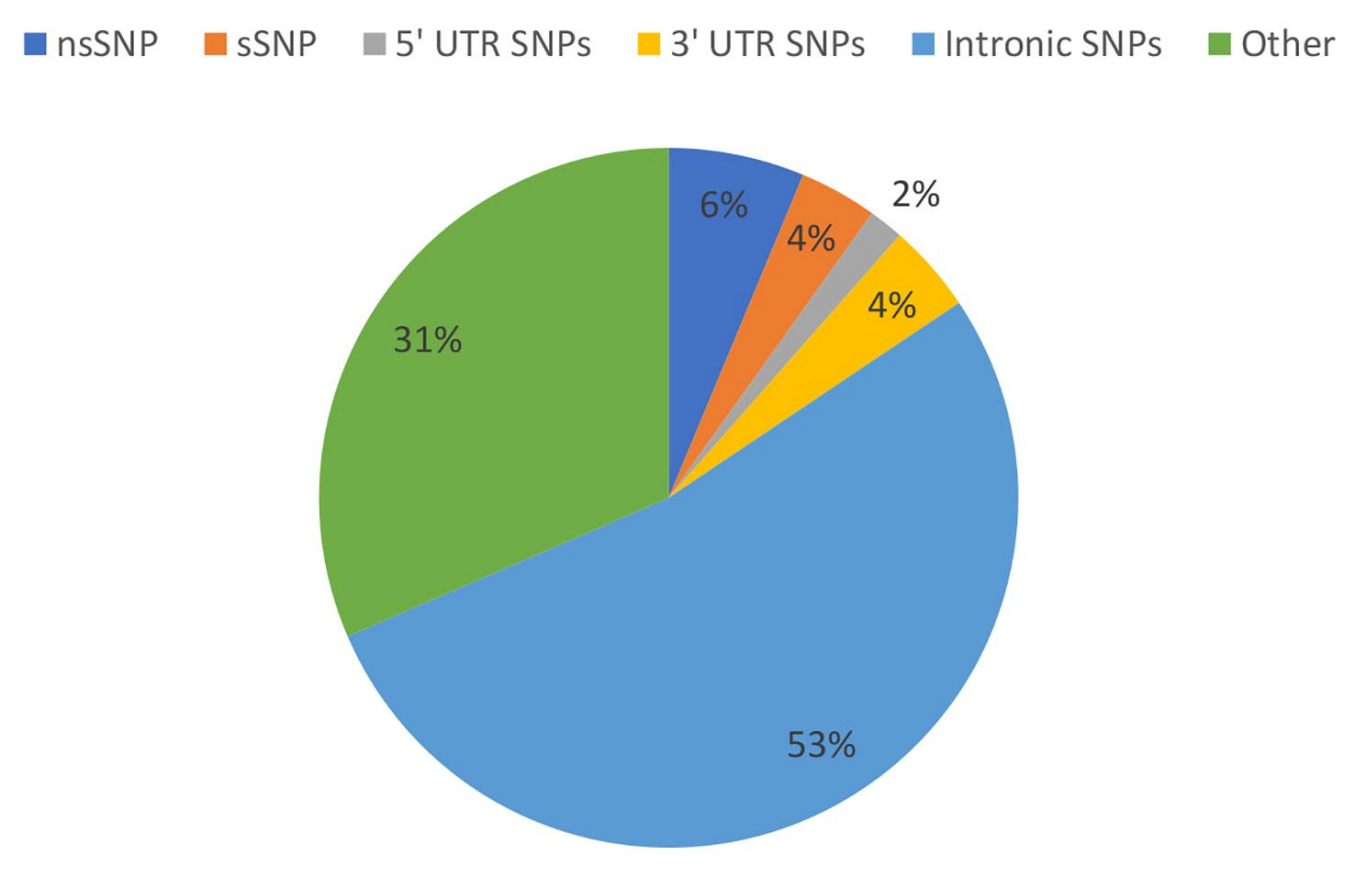
The pie chart shows the percentages of the SNPs in the *IL1B* gene based on the NCBI dbSNP database

The dbSNP database, known for its vast collection of SNPs, was used to retrieve SNPs of interest. A total of 3533 SNPs were retrieved, of which 222 (6%) were nsSNPs, 127 sSNPs (4%), 57 SNPs occurred in 5’UTR (2%), 145 SNPs in 3’UTR (4%), 1870 intronic SNPs (53%), and the rest were of other types (31%) (Fig. 1).

### Deleterious nsSNPs

The impact of 222 nsSNPs on the structure and function of the IL1B protein was investigated using six different in silico prediction algorithms. The accession numbers of 222 nsSNPs are provided in the Supplementary file: Table S1. These tools included SIFT, PolyPhen-2, PANTHER, PROVEAN, PhD-SNP, and SNPs&GO. The high-risk nsSNPs were first assessed using SIFT, PolyPhen-2, and PROVEAN. Subsequently, the nsSNPs showing significant damaging effects were further analyzed for potential disease associations using three online servers (PANTHER, PhD-SNP, and SNPs&GO).

**Fig. 2:**
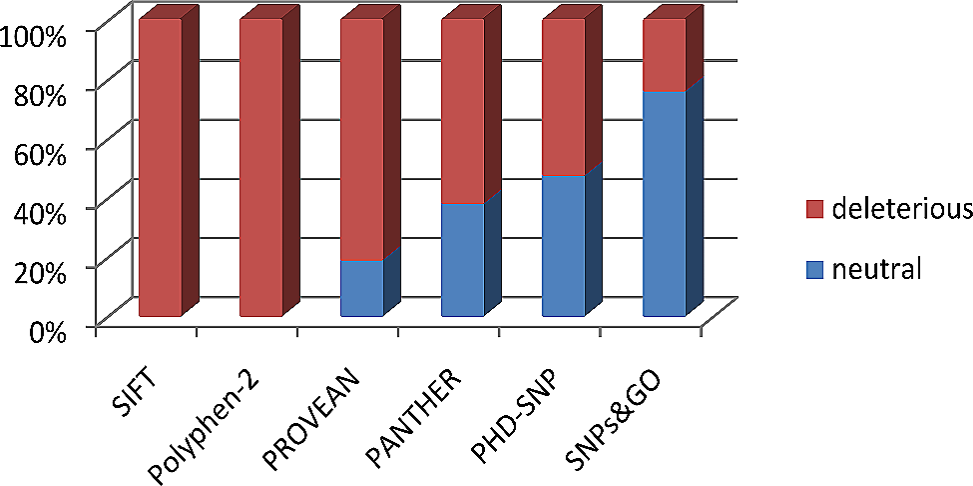
A graphical representation showing the distribution of 21 nsSNPs predicted as deleterious (in red) and neutral (in blue) by six online bioinformatic servers. The 21 nsSNPs were predicted to be damaged by SIFT and PolyPhen-2

Among 222 nsSNPs analyzed by SIFT, 90 nsSNPs were identified to be affecting the protein function with a tolerance index score ≤ 0.05. While 26 nsSNPs showed a highly damaging tolerance index score of 0.00. Subsequently, Polyphen-2 program was also used to identify the damaging SNPs, HumDiv score revealed that 116 nsSNPs were damaging with a score ≥ 0.5, among those 44 nsSNPs scored 1 and were found to be highly damaging. Importantly, 21 of the nsSNPs were predicted to be highly damaged by SIFT (index score of 0) and PolyPhen-2 (index score of 1). Therefore, these 21 SNPs were further investigated by PANTHER, PROVEAN, PhD-SNP, and SNPs&GO (Fig. 2). However, 17 (80.9%) of the 21 nsSNPs were found deleterious. Then, the association of these nsSNPs with diseases was analyzed using PANTHER, PhD-SNP and SNPs&GO. PANTHER indicated that 13 nsSNPs (61.9%) were associated with diseases, while PhD-SNP predicted 11 nsSNPs (52.3%) to be linked to diseases. In contrast, SNPs&GO showed a lower number, identifying only five nsSNPs (23.8%) as disease-associated, see Fig. 2 and (supplementary file; Table S2) for more illustration. Upon integration of the results from the six computational in silico tools mentioned above, it was observed that the predictions overlapped, indicating unanimous agreement on the outcomes [16, 17]. As a cumulative outcome of all the tools, five nsSNPs (E100K, T240I, S53Y, D128Y, F228S) were identified as the most damaging nsSNPs (as shown in Tables 1 and 2). Consequently, all further investigations were focused solely on these five nsSNPs.

**Table 1:** The five most deleterious nsSNPs were investigated by SIFT, PolyPhen-2, and PROVEN servers

ID of SNPs	SUB	SIFT		PolyPhen-2 HumDiv^1^		PolyPhen-2 HumVar^2^		PROVEN	
		Pred	TI	Pred	Sc	Pred	Sc	Pred^*^	Sc
rs1449638481	S53Y	deleterious	0	damaged	1	damaged	0.99	deleterious	-4.235
rs1041942016	E100K	deleterious	0	damaged	1	damaged	1	deleterious	-3.211
rs1478935996	D128Y	deleterious	0	damaged	1	damaged	0.992	deleterious	-8.314
rs1199067235	T240I	deleterious	0	damaged	1	damaged	1	deleterious	-5.518
rs1681958816	F228S	deleterious	0	damaged	1	damaged	0.997	deleterious	-4.656

SUB; amino acid substitutions, Pred; prediction, Sc; score, ^*^cutoff= -2.5

^1^HumDiv: Mendelian disease variants vs. divergence from close mammalian homologs of human proteins ( > = 95% sequence identity)

^2^HumVar: all human variants associated with some disease (except cancer mutations)

**Table 2:** Prediction of the pathogenicity for the five most deleterious nsSNPs using three web servers

ID of SNPs	SUB	PHD-SNP			PANTHER			SNPs&GO		
		Sc	Pred	RI	Sc	Pred	RI	Sc	Pred	RI
rs1449638481	S53Y	0.765	disease	5	0.761	disease	5	0.666	disease	3
rs1041942016	E100K	0.782	disease	6	0.552	disease	1	0.708	disease	4
rs1478935996	D128Y	0.739	disease	2	0.969	disease	9	0.631	disease	3
rs1199067235	T240I	0.74	disease	5	0.643	disease	3	0.537	disease	1
rs1681958816	F228S	0.677	disease	4	0.57	disease	4	0.531	disease	1

SUB; amino acid substitutions, Pred; prediction, Sc; score, RI; reliability index

### Prediction of the stability for the deleterious nsSNPs

To predict any stability alterations in the IL1B protein, we submitted the 21 nsSNPs to I-mutant v2.0, and MUPro to investigate their effect on protein stability. The I-Mutant v2.0 predicted their RI and free energy change values and revealed that four of the SNPs decreased the protein stability while the S53Y SNP increased the stability. On the other hand, MUPro found that all five SNPs decreased the IL1B protein stability (Table 3).

**Table 3:** Prediction of the stability for the five deleterious nsSNPs using I-Mutant v2.0 and MUPro servers

ID of SNPs	SUB	I-Mutant v2.0			MUPro	
		Prediction	RI	DDG	Prediction	Score
rs1449638481	S53Y	increase	3	0.43	decrease	-1.3227
rs1041942016	E100K	decrease	9	-2.61	decrease	1.2026
rs1478935996	D128Y	decrease	2	1.06	decrease	-0.2066
rs1681958816	F228S	decrease	8	-2.43	decrease	-1.1535
rs1199067235	T240I	decrease	5	0.09	decrease	0.0428

DDG; free energy change value, SUB; amino acid substitution, RI; Relatability index

### Conservation profile of the deleterious nsSNPs

It is evident that, compared to those in non-conserved regions, SNPs located in conserved regions were highly damaging. Therefore, we utilized the Consurf server to analyze the conservancy degree of the five nsSNPs of interest. Consurf revealed that all five SNPs scored 9, indicating high conservation. When the residues are highly conserved and exposed, they are predicted as functional importance. Conversely, when they are highly conserved and buried, they are predicted to have structural importance. Remarkably, E100K, T240I, and D128Y were identified with functional importance, and S53Y and F228S with structural importance (Table 4).

**Table 4:** Conservation profile of the five most deleterious nsSNPs using ConSurf server

SNPs ID	Residue and position	Conservation color	Prediction
rs1449638481	S53	9	Highly conserved and buried (s)
rs1041942016	E100	9	Highly conserved and exposed (f)
rs1478935996	D128	9	Highly conserved and exposed (f)
rs1681958816	F228	9	Highly conserved and buried (s)
rs1199067235	T240	9	Highly conserved and exposed (f)

(f); predicted to be functional residue, (s); predicted to be structural residue

### Prediction of the effect of the high-risk nsSNPs on IL1B protein properties

Project Hope was used to predict the effects of the five most deleterious nsSNPs on amino acid size, charge, hydrophobicity, conservancy, structure, and function. It revealed that, for four SNPs, the mutant residue was bigger than the wild-type residue. This might lead to bumps, and in some cases, the bigger mutant residue does not fit in the protein core. For F228S, the mutant residue was smaller than the wild-type residue. So, this mutation may cause an empty space in the core of the protein.

It also showed that in the case of T240I and D128Y, there is a difference in charge between the wild-type and mutant amino acids. In T240I, the wild-type residue charge was negative while the mutant residue charge was positive. While with D128Y, the wild-type residue charge was negative and the mutant residue charge was neutral. This difference in charge will affect the ionic interaction made by the original wild-type residue. In addition, two mutations (T240I, D128Y) were found to be changing the hydrophobicity of the amino acid where the mutant residue was more hydrophobic than the wild-type residue. In T240I the difference in hydrophobicity may affect hydrogen bond formation. Project Hope also provided conservancy results and they were the same as Consurf. All the wild-type residues were found to be very conserved and probably affecting the protein structure or function.

### 3D structural analysis

**Fig. 3:**
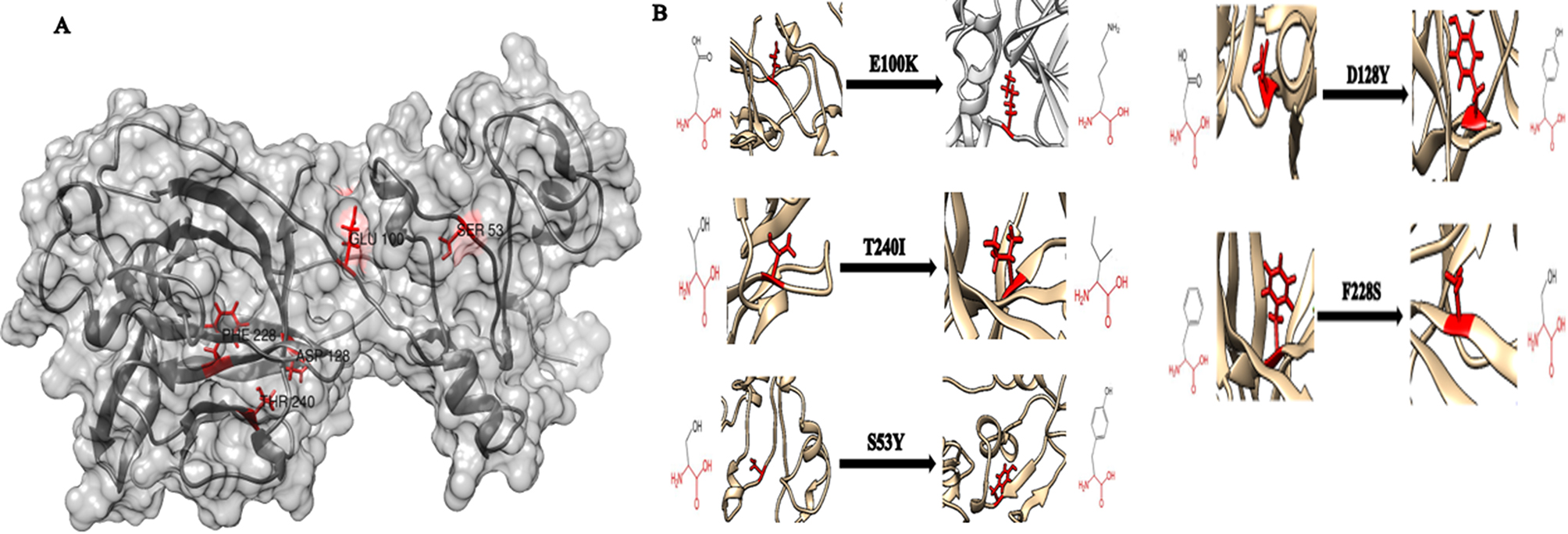
(**A**) 3D structure of the native IL1B protein. (**B**) Effect of the five most deleterious nsSNPs on the IL1B protein structure. Chimera software was used to visualize the 3D structure

Project Hope, RaptorX, and UCSF Chimera were employed to predict and visualize the 3D structure of mutated models of the IL1B protein. RaptorX generated a 3D structure model for IL1B, allowing the mapping of amino acid substitutions. These models were then visualized using UCSF Chimera (Fig. 3).

### Prediction of cancer-causing nsSNPs

**Fig. 4:**
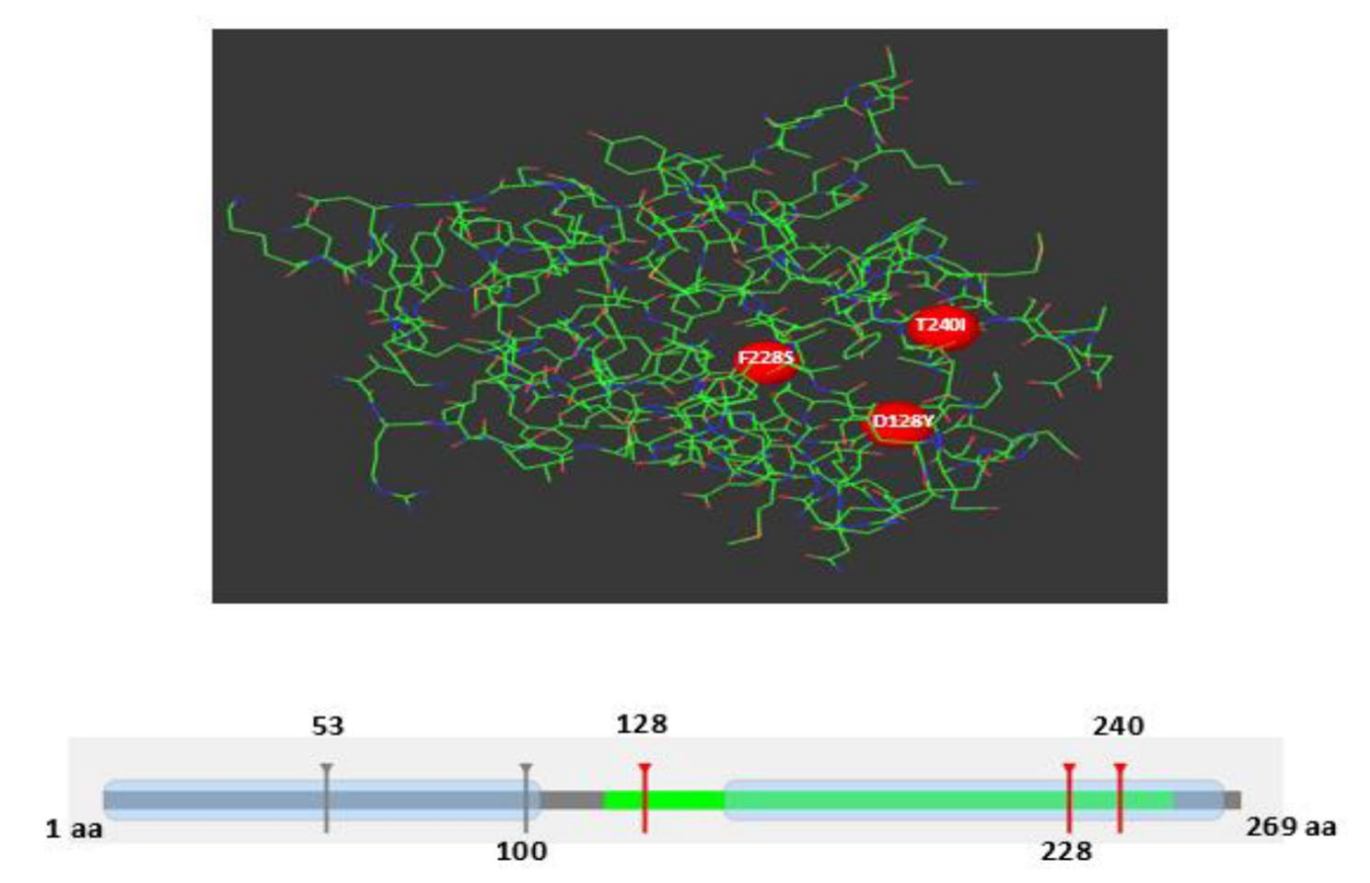
Prediction of three cancer-causing nsSNPS (colored red) in IL1B protein using Mutation 3D server

Mutations in the IL1B protein can result in structural and functional alterations that might lead to tumor formation. Therefore, the Mutation 3D server is utilized to predict harmful nsSNPs associated with cancer development. The analysis identified D128Y, F228S, and T240I as mutations associated with cancer (colored red), while S53Y and E100K are uncovered mutations (colored gray), see Fig. 4.

### Molecular dynamics, structural stability, and flexibility analysis

**Fig. 5:**
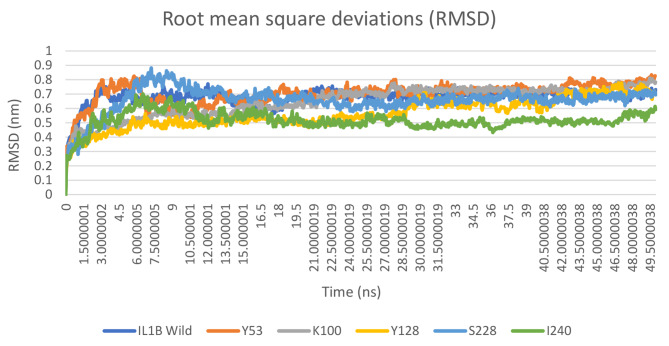
The RMSD values of wild type (blue) and five mutant structures of IL1B protein

Molecular dynamic (MD) simulations were conducted to examine how the IL1B protein undergoes atomic-level changes over time. The simulations were run for 50 ns. The impact of mutations on the protein stability was assessed using root mean square deviation (RMSD) values. These values represent the extent to which the protein’s backbone atoms deviate from their initial structure and serve as a key indicator of the protein system’s convergence. The RMSD values for both the original and mutant models were calculated based on the trajectory files. As illustrated in Fig. 5. The native structure IL1B average RMSD is approximately 0.68 nm which was increased in mutant S53Y (an average of ∼ 0.7 nm) and decreased in other mutants, ranging from about 0.52 to 0.66 nm. This difference in the range of deviation observed in the mutant model reflects the change in protein stability and elucidates the effect of the mutated amino acid on the protein structure.

**Fig. 6:**
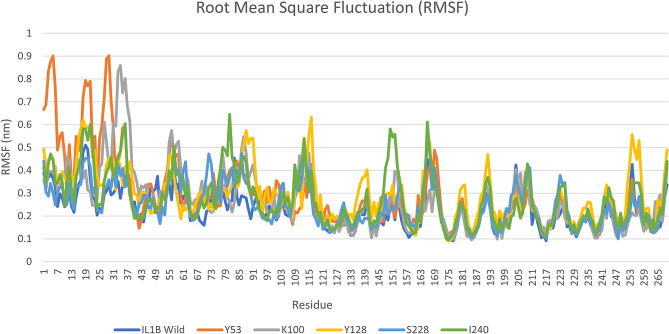
The RMSF values of wild type (blue) and five mutant structures of IL1B protein

To assess the structural flexibility of both the native and mutant IL1B protein models, we calculated the root mean square fluctuation (RMSF) values using data from a 50 ns simulation trajectory. The RMSF values for the IL1B native and mutant models are presented in Fig. 6. The highest residual fluctuation for the native structure was 0.5099 nm, 0.4909 nm, and 0.4798 nm noticed at the positions of Glu 19, Asp 20, and Asn 18, respectively. Overall, RMSFs of all the mutant models deviated considerably from the native structure in the entire simulation period. Among them, mutant S53Y exhibited the highest residual fluctuation. A change in the RMSFs indicates alterations in the mode of flexibility in the mutant models, reflecting the impact of deleterious amino acid substitutions in the IL1B protein.

### Effects of deleterious mutations in hydrogen bonding, solvent accessible surface area, structural rigidity, and electrostatic potential of IL1B protein

**Fig. 7:**
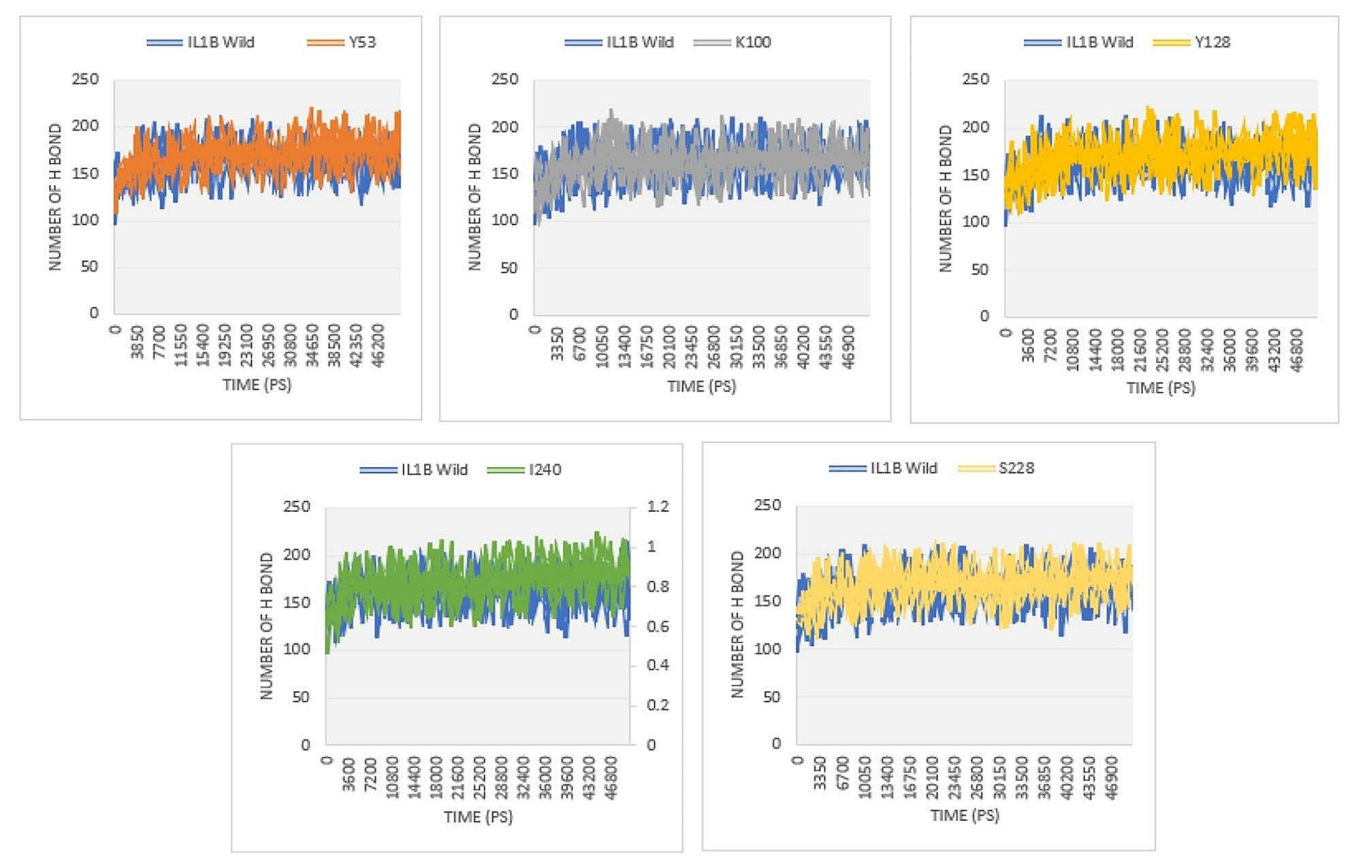
The number of hydrogen bonds formed in wild type and mutant structures of IL1B protein

Hydrogen bonds play pivotal roles in determining the stability of proteins. nsSNPs can influence the functionality of the native protein by impacting the formation of hydrogen bonds [18]. Figure 7 illustrates the number of hydrogen bonds formed in both the native and mutant structures of the IL1B protein. The native structure of IL1B protein exhibits an average number of ∼ 162 hydrogen bonds ranging from 122 to 188 hydrogen bonds throughout the 50 ns simulation period. Mutant model E100K obtained a closer number of hydrogen bonds, about 155 to 192 in comparison with the native structure. While the remaining mutant models (S53Y, D128Y, F228S, and T240I) exhibited a higher number of hydrogen bonds, with an average of approximately 170, 170, 168, and 172 hydrogen bonds throughout the 50 ns simulations (Fig. 7).

**Fig. 8:**
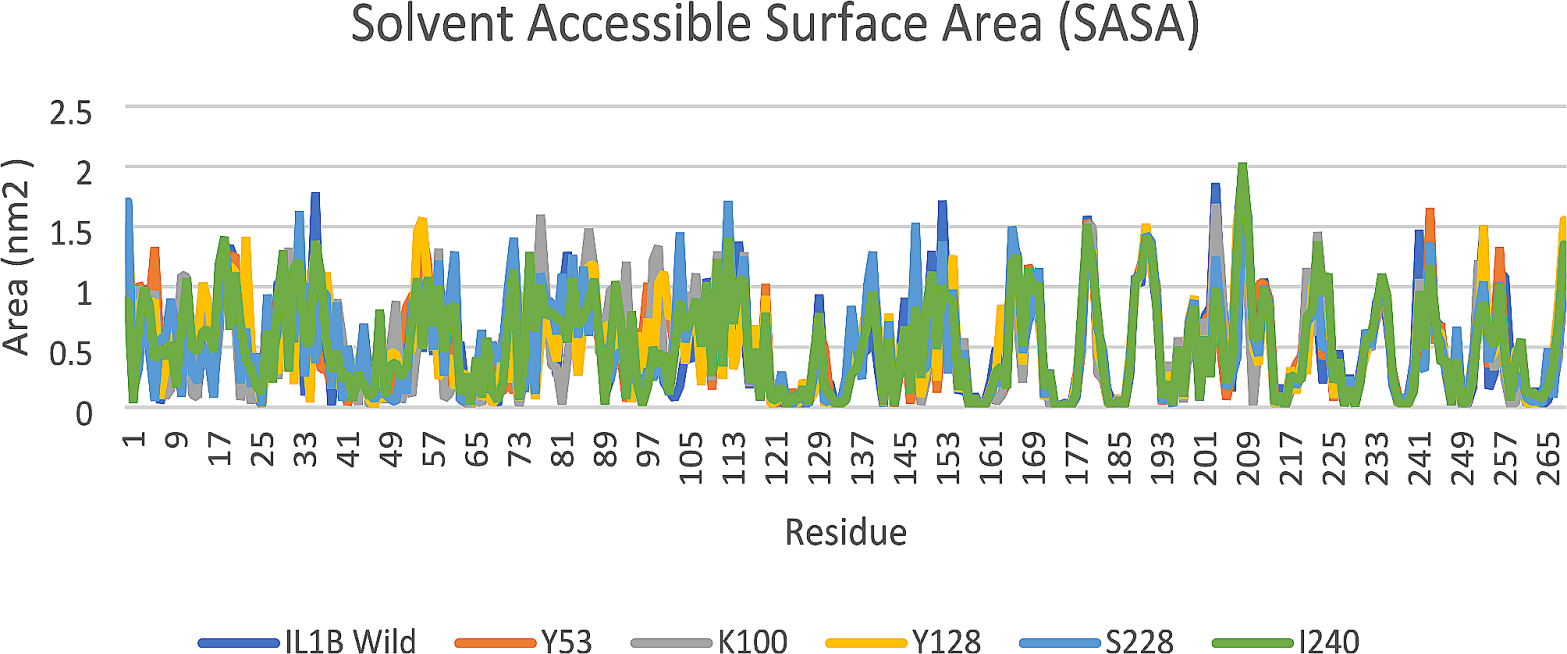
The SASA values of wild type and mutant structures of IL1B protein

Afterward, we assessed the solvent accessible surface area (SASA) and found that the SASA values of native and mutant proteins varied greatly during the 50 ns simulations period. The IL1B native structure average SASA value was ∼ 0.52 nm^2^ and the highest fluctuation was seen at the position of Lys 209. Mutant D128Y exhibited a SASA value similar to that of the native structure. In contrast, S53Y and T240I exhibited greater SASA values, while E100K and F228S showed lower average SASA values compared to the native structure (Fig. 8).

**Fig. 9:**
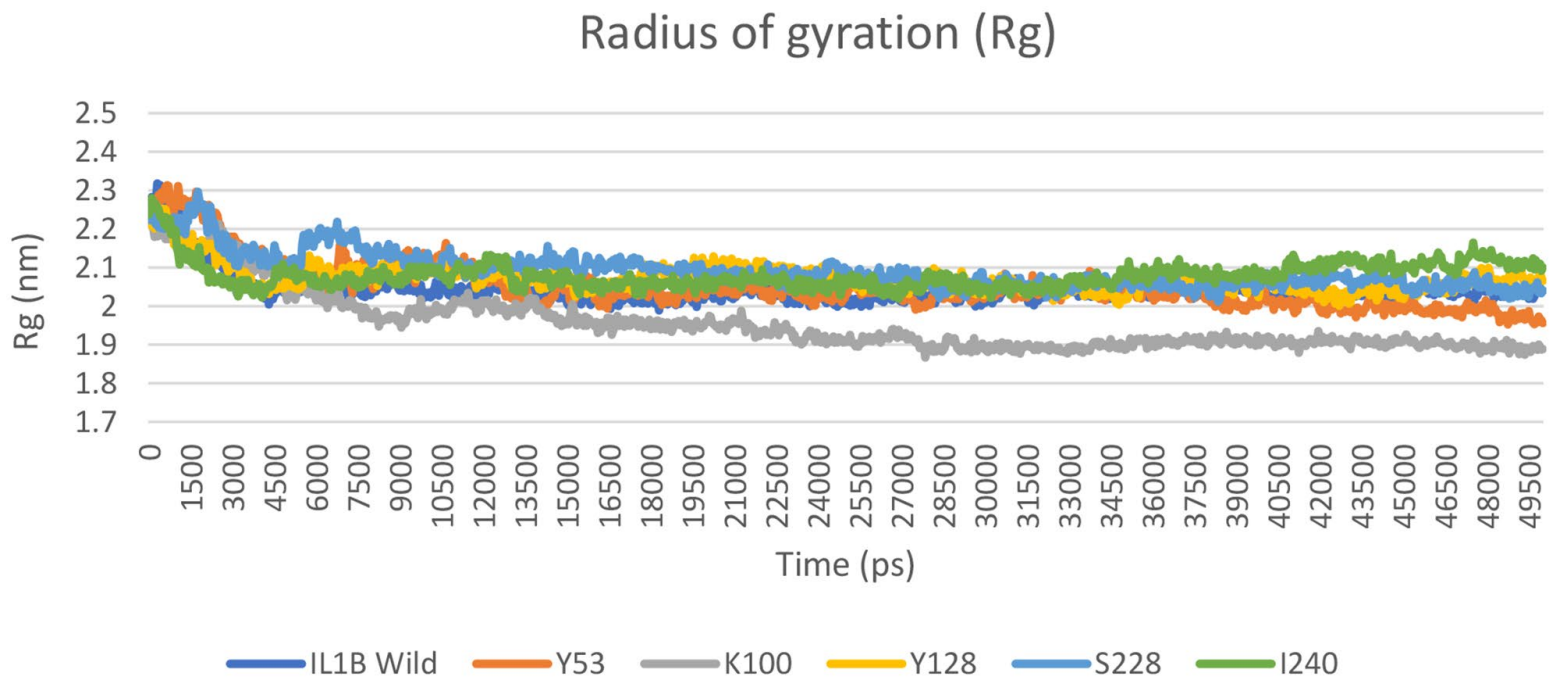
The *Rg* values of wild type and mutant structure of IL1B protein

Moreover, we calculated the radius of gyration (*Rg*) to assess the compactness and rigidity of the native and mutant structures of the IL1B protein. The average *Rg* value of the wild structure was ~ 2.04 nm and ranged from ∼ 1.99 nm to ∼ 2.31 nm. All the mutants exhibited fluctuation of *Rg* values ranging from ∼ 1.87 nm to ∼ 2.31 nm, as illustrated in Fig. 9. S53Y, E100K, D128Y, F228S, and T240I average *Rg* values were ∼ 2.05 nm, ∼ 1.95 nm, ∼ 2.07 nm, ∼ 2.09 nm, and ∼ 2.08 nm, respectively.

### *IL1B* function and gene interactions

**Fig. 10:**
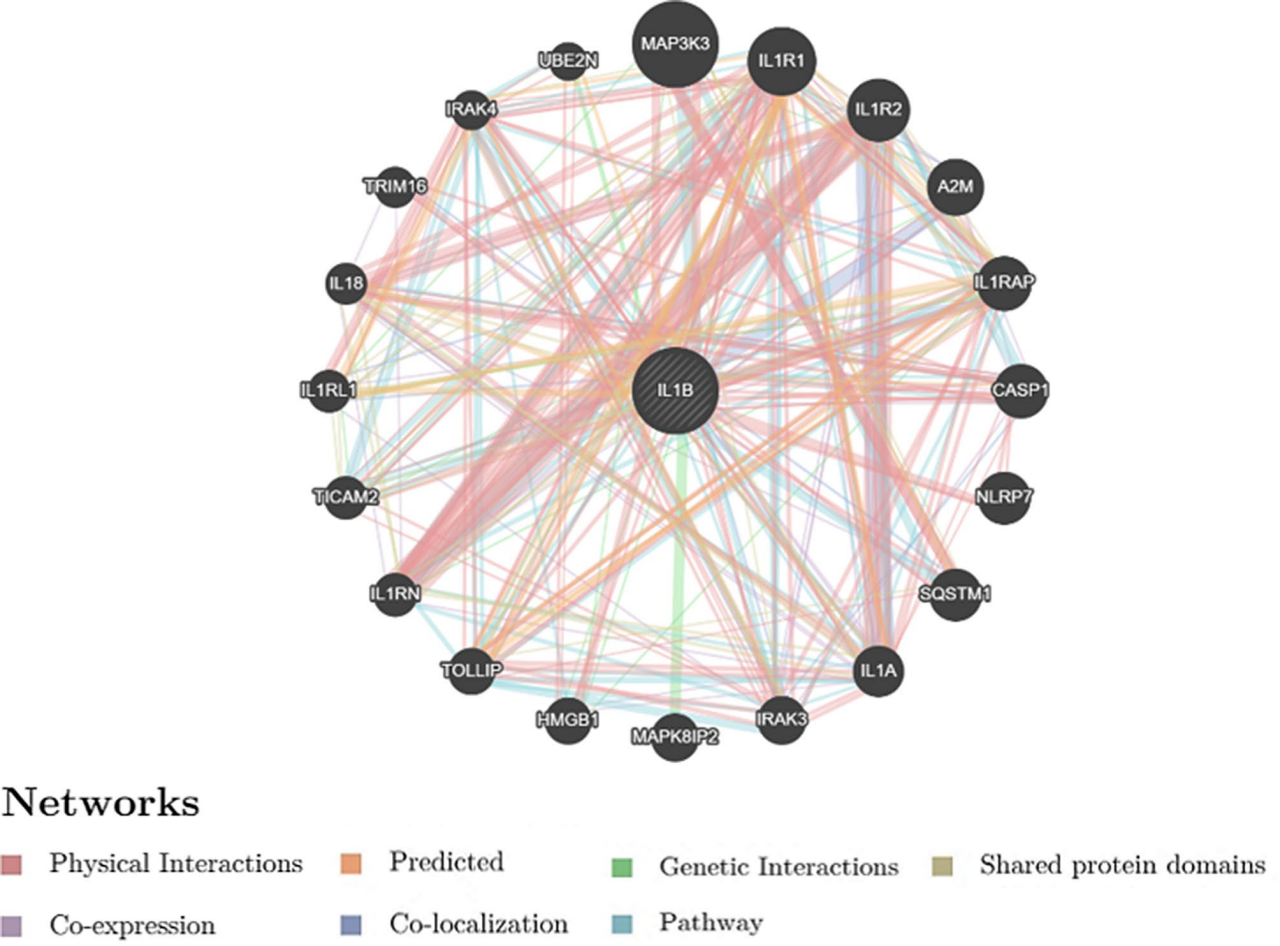
*IL1B *gene network interactions predicted by GeneMANIA web server

GeneMANIA was used to identify protein function and to predict *IL1B* gene interactions and network (Fig. 10). It revealed that *IL1B* has many vital functions such as response to interleukin-1, CD4-positive, alpha-beta T cell cytokine production, regulation of T cell-mediated immunity, regulation of I-kappaB kinase/NF-kappaB signaling, cellular response to molecule of bacterial origin and leukocyte cell-cell adhesion. An overview of *IL1B* gene functions and its network interactions is illustrated in the supplementary file; Table S3. The associated genes that contribute to *IL1B* to accomplish its function are presented in the supplementary file; Table S4.

## Discussion

IL1β is known to play a central role in infectious and inflammatory diseases. Most genetic analyses related to *IL1B* have focused on the promoter, neglecting the coding region. For instance, the two SNPs (CT; dbSNP: rs16944) and (TC; dbSNP: rs1143627) are located upstream at positions − 511 and − 31, respectively. These polymorphisms in the *IL1B* gene have been found associated with several inflammatory diseases, infectious diseases, autoimmune disorders, depression, and several types of cancers such as hepatocellular carcinoma, lung cancer, breast cancer, gastric cancer, and lymphoblastic leukemia [9, 11, 14, 19–21]. In the coding region of IL1B, the SNP (CT; dbSNP: rs1143634) at position + 3954 in exon5 has been associated with several diseases, such as aseptic prosthetic loosening, prosthetic joint infections, endometriosis, and cancers [22–24]. Generally, silent SNPs can lead to the production of truncated and inactive proteins due to their impact on splicing sites and premature mRNA transcription termination. But in the case of rs1143634, this polymorphism increases the concentration of active IL-1B rather than inactive protein. The excess amount of IL-1B promotes an environment conducive to cancer development by enhancing uncontrolled cellular proliferation and differentiation while interfering with apoptosis [24].

In this study, we addressed a critical gap by predicting the functional consequences of 222 nsSNPs with 199 rs IDs located in the coding region, shedding light on how variations in the coding regions of *IL1B* may contribute to disease susceptibility and progression. It is therefore critical to identify deleterious nsSNPs in the *IL1B* gene, as these specific mutations have the most substantial impact on protein structure and function, which directly contribute to disease pathologies [25]. Six web servers (SIFT, PolyPhen-2, PROVEAN, SNPs & GO, PHD-SNP, PANTHER) were used to analyze the impact of missense SNPs on the function and structure of IL1B protein; then the results of the above servers were integrated [26]. Consequently, in silico algorithms predicted five nsSNPs, (rs1041942016; E100K), (rs1199067235; T240I), (rs1449638481; S53Y), (rs1478935996; D128Y), (rs1681958816; F228S), that were highly deleterious based on their compared prediction scores (Tables 1 and 2).

In addition, I-mutant v2.0, and MUPro were used to predict the stability alterations in the IL1B protein. I-Mutant v2.0 revealed that four of the SNPs decreased the protein stability while S53Y SNP increased it, however, MUPro found that all five SNPs decreased the stability. According to the literature, decreased protein stability can lead to an increase in degradation, aggregation, and misfolding of proteins [27, 28]. In contrast, missense variations can increase protein stability but remain deleterious [29]. Additionally, a mutation can result in an altered function rather than a loss of function. These altered functions might contribute to the development or progression of diseases like cancer. In the case of proinflammatory cytokines, which are associated with inflammation and immune responses, a destabilizing nsSNP that alters their function could potentially lead to aberrant immune responses and contribute to the development of diseases, including cancer, especially in the context of inflammation-driven cancers. For instance, the A114S nsSNP in the *IL1A* gene (GT; dbSNP: rs17561) is predicted to decrease protein stability and has been strongly associated with various types of diseases that affect inflammatory responses, atopy, and infectious diseases, such as malaria [5, 30, 31]. Also, Dakal et al. predicted a number of deleterious nsSNPs in *IL-8* which destabilize the protein and have a potential role in IL-8 binding to its receptors on inflammatory cells and in disease susceptibility [4].

Generally, conserved residues are more likely to be damaging and involved in controlling the biological system in proteins, such as folding and/or stability [32]. However, functional amino acids are located at binding and catalytic sites and display substantial protein-protein interaction [33]. In this study, we assessed the evolutionary conservation profile of the five nsSNPs positions in IL1B protein via ConSurf web server. This server predicted that all the five nsSNPs were highly conserved with a score of nine and classified E100K, T240I, and D128Y mutations as highly conserved and exposed (functional), while S53Y and F228S were classified as highly conserved and buried (structural). Structural mutations affect buried residues in the protein core, resulting in changes in amino acid charge and size, salt bridges, S–S bridges, and hydrogen bonds. These changes could alter the structure and function of the protein [34].

In this study, Project Hope software predicted that the five deleterious nsSNPs are positioned within an essential domain responsible for binding to other molecules, and the mutations in these residues could potentially modify this vital function. Furthermore, the variations in mass and charge between the wild-type and mutant residues within the protein have an impact on the spatiotemporal dynamics of protein-protein interactions [35, 36]. In the present study, Project Hope analysis revealed distinct differences in charge and hydrophobicity between the wild-type and mutant amino acids for the T240I and D128Y mutations. Typically, the change in hydrophobicity is known to influence hydrogen bond formation and interactions between the residues [37]. As a result, these nsSNPs could lead to a potential dysregulation in the expression of IL1B and inflammatory responses which could contribute to the development and progression of inflammatory diseases and cancers. The 3D structure of IL1B was predicted by RaptorX and modeled using UCSF Chimera to compare the differences between the wild and the mutant amino acids. As illustrated in Fig. 3, these mutations alter the original conformation of the native protein.

The significance of the IL1B and its role in the immune system was previously described, for a review see [21, 38]. In this study, GeneMANIA software was used to identify protein function and to predict *IL1B* gene interactions and network. Some of these functions were T cell differentiation, leukocyte proliferation, regulation of leukocyte cell-cell adhesion, regulation of T cell-mediated immunity, regulation of I-kappaB kinase/NF-kappaB signaling and cellular response to molecule of bacterial origin, see Fig. 10 and Table S3 and Table S4.

However, polymorphisms in *IL1B* play a role in depression, multiple inflammatory-associated disorders including thyroid disease, arthritis, and septic shock, and susceptibility and response to infection [9, 21, 39]. In addition, mutations in the *IL1B* gene have emerged as intriguing factors in the landscape of cancer development and progression. These genetic alterations can lead to dysregulation of the intricate balance between inflammation and immune response within the tumor microenvironment [14]. As research continues to unravel the complexities of *IL1B* mutations and their implications, the intersection between genetic variants in this cytokine gene and oncogenesis offers a promising avenue for developing novel therapeutic strategies aimed at modulating the inflammatory processes that drive cancer progression. In this study, among the five most deleterious nsSNPs, we identified three cancer-causing nsSNPs (D128Y, F228S, and T240I) which are clustered in one domain (Fig. 4). D128Y is located in loop 9 which is important for receptor binding and bioactivity of IL1B [40]. Also, a mutation of aspartic acid (D) to tyrosine (Y) could potentially result in an overall increase in the positive charge of the IL1B protein. The positive charge of IL1B allows it to bind to negatively charged phosphatidylinositol 4,5-bisphosphate (PIP2) in the cell plasma membrane. Subsequently, the release of microvesicles from the plasma membrane facilitates the exit of IL-1B from the cell [41]. On the other hand, F228S, and T240I are located in the motif (228aa − 241aa) which is involved in the interaction with TMED10. TMED10 is a protein channel that regulates the secretion of a broad spectrum of cytosolic proteins lacking a signal peptide, including inflammatory factors (IL-1 family members) [42]. Because these mutations are located in critical positions, nsSNPs might have similar functional consequences as seen in the case of IL-1B deregulation. This implies that these three nsSNPs could potentially play a role in the development of diseases, such as inflammatory diseases and cancers. Moreover, E100K mutation provides another surface-exposed Lys residue in IL1B which mediates integrin binding. Previous mutagenesis studies found that the E105K and E128K mutations in IL1R-binding sites in IL-1β enhance integrin binding [43, 44]. Integrin binding is essential for IL-1B signaling and involved in its agonistic action [45].

To gain insight into the protein structure and behavior under physiological conditions and to understand the detrimental effects caused by these mutations, we conducted Molecular Dynamics (MD) analysis on the IL1B protein. In the 50 ns simulation trajectory, we observed variations in the values of mutants compared to the wild type in terms of RMSD, RMSF, *Rg*, SASA, and the number of hydrogen bonds. The results from the MD simulations provide valuable information regarding the changes occurring in the structures of native and mutant IL1B proteins under physiological conditions. In this study, changes in molecular stability and flexibility were identified through the analysis of RMSD and RMSF. Stability is a key attribute that plays a pivotal role in influencing the function, activity, and regulation of biomolecules. The native structure of IL1B exhibits an average RMSD of approximately 0.68 nm. This value increased in the mutant S53Y (averaging ∼ 0.7 nm) and decreased in other mutants, ranging from about 0.52 to 0.66 nm. These results indicate that the protein stabilities of E100K, D128Y, F228S, and T240I exhibit lower levels of deviation compared to both the native and S53Y proteins. The larger the deviations, the less stable the protein structure [46]. From the fluctuation analysis, the flexibility of all mutant models of IL1B protein is heterogeneous in comparison with the native protein, as measured by RMSF, see Fig. 6. Increasing flexibility can render the protein more flexible, while a decrease in flexibility can make the protein more rigid. However, conformational changes are essential for various protein functions, but achieving a proper balance between conformational flexibility and rigidity is crucial [18].

The hydrogen bonds serve as the main contributors in maintaining the protein’s structural conformation [47]. From the hydrogen bond analysis, we found that the number of hydrogen bonds in all mutant structures is higher than in the native structure. Additional hydrogen bonds can strengthen the interactions between amino acids and other molecules, such as ligands or other protein subunits [48]. However, it is important to note that the specific consequences of gaining hydrogen bonds in a mutant protein structure will vary depending on the protein’s function, its cellular environment, and the specific location and nature of these bonds. Experimental studies are required to assess the effects of these mutations on protein structure and function. In addition, the SASA analysis also effectively demonstrates the influence of mutations on the IL1B structure. Changes in surface area can potentially affect ligand binding and protein stability [47]. We also calculated the *Rg* to assess the overall dimensions of the protein [49]. As shown in Fig. 9, the mutant proteins exhibited a level of compactness closer to the native IL1B structure, except for E100K which had a lesser *Rg*. Differences in the level of *Rg* compared to the native protein suggest structural and conformational changes resulting from the mutation.

The primary drawback of this study is that the tools employed in this work to screen out deleterious nsSNPs are based mainly on a computational approach. Therefore, in vitro and in vivo genotype/phenotype correlation studies are recommended. Additionally, research on the frequency of these mutations across different geographical locations is necessary. Disease prediction methods across various in silico tools share similarities, largely based on changes in conserved residues over time. Despite these limitations, this study serves as a foundation for future large-scale research on investigating the relationship between IL1B nsSNPs, diverse diseases, and potential therapeutic interventions.

## Conclusions

In this study, five nsSNPs (E100K, T240I, S53Y, D128Y, and F228S) were found to be deleterious and have a mutational impact on the structure and function of the IL1B protein. These nsSNPs can be considered significant candidates in the pathogenesis of disorders and cancers caused by IL1B dysfunction, contributing to effective drug discovery and the development of precision medications. Thorough research and wet lab experiments are required to confirm the deleterious effect of these polymorphisms on protein structure and function. Moreover, the computational approach was found valuable in predicting deleterious nsSNPs.

## Methods

### Data mining

Data regarding the human *IL1B* gene was obtained from the National Center for Biotechnology Information (NCBI) website, which serves as the largest repository for SNP data, encompassing more than 140 million submitted genetic variations. The specific SNP details, including the protein accession number and SNP ID, were extracted from the NCBI dbSNP (http://www.ncbi.nlm.nih.gov/snp/) database. The accession numbers of all downloaded data of nsSNPs from NCBI are provided in the Supplementary file: Table S1.

### Prediction of deleterious nsSNPs

In this study, six web servers were used to predict the functional impact and pathogenic nature of nsSNPs. If not stated otherwise, all tools were used according to their default settings.

#### Sorting intolerant from tolerant (SIFT) SIFT

SIFT (https://sift.bii.a-star.edu.sg/) is a predictive method that relies on sequence homology. It distinguishes between intolerant and tolerant amino acid substitutions and evaluates the functional impact of SNPs [50]. The prediction is based on the conservation level of each amino acid residue in the query sequence. SIFT creates a dataset of functionally related protein sequences and then computes a normalized probability for each replacement at every position in the alignment. These probabilities are stored in a scaled probability matrix, also known as the SIFT score. A SIFT score below 0.05 indicates that a missense variant is likely to be harmful, while a score greater than or equal to 0.05 suggests that the variant is likely benign.

#### Polymorphism phenotyping v2 (PolyPhen-2)

PolyPhen-2 (http://genetics.bwh.harvard.edu/pph2/) is a web tool designed to assess the effects of amino acid substitutions on human protein structure and function. It utilizes multiple sequence alignment and protein 3D structure analysis for its predictions [51]. It also calculates position-specific independent count scores (PSIC) for each of two variants, and then calculates the PSIC scores difference between two variants. The greater the PSIC score difference, the greater the functional impact of a certain amino acid alteration. A score of (0.96–1) is regarded ‘Probably damaging,’ while a score of (0.71–0.95) is considered ‘Possibly damaging’ and a score of (0.31–0.7) is considered ‘benign’.

#### Protein variation effect analyzer (PROVEAN)

PROVEAN (http://provean.jcvi.org/index.php) is a predictive algorithm capable of evaluating the effects of single and multiple amino acid substitutions, insertions, and deletions [52]. To utilize the server, one must input the protein sequence and the amino acid variants. The algorithm then conducts a BLAST search to identify homologous sequences and generates corresponding scores. A final score below 2.5 indicates that a variant is likely to be ‘deleterious,’ while a score equal to or above 2.5 suggests that the variant is likely to be ‘neutral’.

#### Predictor of human deleterious single nucleotide polymorphisms (PhD-SNP)

PhD-SNP (http://snps.biofold.org/phd-snp/phd-snp.html) is a software tool designed to forecast the impact of amino acid substitutions or indels on a protein’s biological function. It aids in identifying non-synonymous or indel variants that are likely to have functional significance by filtering through sequence variants.

#### Single nucleotide polymorphism database and gene ontology (SNPs&GO)

SNPs&GO (http://snps.biofold.org/snps-and-go/snps-and-go.html) is a support vector machine (SVM)-based method used to predict disease-related mutations within protein sequences. The approach utilizes information from protein sequence, structure, and function. To make predictions, the tool requires the input of a complete protein sequence in FASTA format, and/or its three-dimensional structure, along with the target SNP and its corresponding functional Gene Ontology (GO) terms. The output provides probabilities for each protein variation to be either disease-related or neutral. The predicted variant score demonstrates 82% accuracy, with a Matthews’ correlation coefficient of 0.63. Variants with a score greater than 0.5 are classified as ‘disease’.

### Analysis of SNPs evolutionary conservation using PANTHER

Protein Analysis through Evolutionary Relationships (PANTHER) (http://www.pantherdb.org/) is a tool utilized for variant analysis [53]. It utilizes statistical methods based on the Hidden Markov Model (HMM) and multiple sequence alignments to generate a substitution position-specific evolutionary conservation score (subPSEC). SNPs with a score less than − 3 are classified as deleterious, while those with a score greater than − 3 are considered neutral.

### Analysis of nsSNPs stability

#### I-Mutant v2.0

I-Mutant v2.0 is a neural network-based tool designed to analyze routine protein stability and changes, with a particular focus on single-site mutations [54]. By utilizing the FASTA sequence of a protein obtained from UniProt as input, it can predict the impact of mutations on protein stability. The tool is accessible at (http://gpcr2.biocomp.unibo.it/cgi/predictors/I-Mutant3.0/IMutant3.0.cgi).

#### MUPro

MUPro is a SVM-based method used to forecast changes in protein stability caused by nsSNPs. It predicts the energy change value and calculates a confidence score ranging from − 1 to 1 to assess the reliability of the prediction. A score of 0 signifies that the variant decreases protein stability, while a score greater than 0 suggests that the variant enhances protein stability. The MUPro software and datasets can be accessed at http://www.igb.uci.edu/servers/servers.html.

### Phylogenetic conservation analysis

The ConSurf web server (http://consurf.tau.ac.il) is employed to analyze the evolutionary pattern of amino/nucleic acids in macromolecules to identify crucial regions for function and/or structure [55]. The conservation score ranges from 1 to 9, with 1 representing rapidly evolving (variable) regions, 5 indicating moderately evolving regions, and 9 indicating highly conserved positions. Exposed residues with high scores are presumed to be functional, while buried residues with high scores are considered to be structural.

### Biophysical and 3D structural analyses of nsSNPs

#### Project hope

Project HOPE version 1.1.1 is an online service dedicated to examining the structural consequences of a point mutation in a protein sequence [56]. It allows users access to explore structural data from several databases, including UniProt. For the analysis of our SNPs of interest, we utilized the FASTA format sequence of the IL1B protein provided by UniProt as input to predict biophysical validation. The primary objective of submitting to Project HOPE was to analyze and validate the data we had acquired earlier.

#### 3D structural prediction and visualization

RaptorX (http://raptorx.uchicago.edu/BindingSite/) is an online platform that utilizes the RaptorX 3D model to predict the binding sites of a protein sequence [57]. RaptorX is specifically designed for predicting tertiary structures in proteins. We used the new version of RaptorX which is distance-based protein folding powered by deep learning. The predicted structures are further visualized using UCSF Chimera version 1.17.1 (https://www.cgl.ucsf.edu/chimera/). Chimera is a robust application that enables interactive viewing and analysis of various molecular structures and related data, including density maps, sequence alignments, supramolecular assemblies, trajectories, docking results, and conformational ensembles [58].

### Prediction of cancer-causing nsSNPs

Mutation 3D (http://www.mutation3d.org/) serves the purpose of identifying clusters of amino acid substitutions resulting from somatic mutations in cancer [59]. This tool proves valuable for studying the spatial arrangement of altered amino acids within protein models and structures. By inputting a specific protein and its associated mutations, the program employs a 3D clustering method to pinpoint amino acid substitutions that potentially contribute to carcinogenesis.

### Molecular dynamics simulation analysis

Molecular dynamics (MD) is an effective method for examining the evolution of molecular systems and predicting their properties based on the interactions within them. In this study, we used the WebGro server (https://simlab.uams.edu/) to perform simulations and assess the stability and flexibility of the predicted structures [60]. The simulations utilized a triclinic periodic box with a simple point charge (SPC) water model to represent the complex system, along with GROMOS96 43a1 force field settings. The electrically neutralized system was obtained by adding 0.15 M salt. Temperature and pressure conditions were set at 300 K and 1.0 bar, respectively. Each simulation consisted of 1000 frames and took approximately 50 ns to complete. Analysis of the simulations involved examining the root mean square deviation (RMSD) of individual atoms and the root mean square fluctuation (RMSF) of amino acid residues. Additionally, we conducted analyses for hydrogen bonds, radius of gyration (*Rg*), and solvent accessible surface area (SASA) to investigate the impact of mutations on the system.

### Analysis of *IL1B* function and interaction using GeneMANIA

GeneMANIA is an online platform that generates hypotheses about gene function, examines gene lists, and prioritizes genes for functional assays. It achieves this by expanding the query list with genes that have similar functions, identified through available genomics and proteomics data. The GeneMANIA prediction algorithm exhibits high accuracy, and its extensive database makes it a valuable tool for biologists. GeneMANIA (updated version 2018) is available at (http://genemania.org/) [61].

### Electronic supplementary material

Below is the link to the electronic supplementary material.

Supplementary Material 1

## Data Availability

The datasets generated or analyzed during this study are available in the manuscript. The accession numbers of all downloaded data of nsSNPs from NCBI are provided in the Supplementary file: Table S1.
